# Resistant dextrin promotes beneficial fecal bacteria in high and low fiber diet populations: a randomized, double-blinded, controlled pilot study

**DOI:** 10.3389/fnut.2026.1810842

**Published:** 2026-05-20

**Authors:** Caroline Perreau, Diego Tomassi, Odile Capronnier, Thomas Carton, Clémentine Thabuis, Laetitia Guérin-Deremaux

**Affiliations:** 1 ROQUETTE, Life Sciences R&D, Lestrem, France; 2 BIOFORTIS SAS, Saint-Herblain, France

**Keywords:** fiber, gut microbiota, *Parabacteroides*, prebiotic, resistant dextrin

## Abstract

**Background and objectives:**

The average intake of dietary fibers known to beneficially modulate the gut microbiota is below recommendations in high-income countries. This study aimed to evaluate the gut effects of resistant dextrin in healthy male adults with normal weight, consuming high (≥ 25 g/day; HF) or low (≤15 g/day; LF) levels of fiber as part of their usual diet.

**Methods:**

In this randomized, double-blind, placebo-controlled trial, participants received either 15 g/day of resistant dextrin (NUTRIOSE^®^ soluble fiber) or a placebo (maltodextrin) for 4 weeks. Gut microbiota composition, and especially the relative abundance of the *Parabacteroides* genus, gut microbiota function, fecal parameters [pH, short chain fatty acids (SCFAs) and secretory IgA], and stool frequency and consistency, were assessed.

**Results:**

Overall, 124 subjects were recruited, 62 in each treatment arm; 57 in HF, 67 in LF; age: 36.2 ± 11.6 years. The mean relative abundance of *Parabacteroides* at baseline was similar in the LF (1.5%) and HF (1.3%) subgroups. It increased with the active supplementation and remained stable with the placebo, both in the entire population (5.5% vs. 1.4%; *p* < 0.0001) and in the subgroups of dietary fiber intake (HF: 5.5% vs. 1.5%; LF: 5.5% vs. 1.3%; *p* < 0.0001), then returned to baseline levels 2 weeks later. Among *Parabacteroides*, *P. distasonis* showed the highest abundance in the active arm as compared to placebo (2.6% vs. 0.5%; *p* < 0.0001). The *Bacillota* phylum was less abundant (tendency) at the end of intervention (55.4% vs. 59.0%; *p* = 0.0545). Conversely, several species from the *Clostridium* genus were more abundant. From a functional perspective, resistant dextrin intake tended to increase bacterial genes involved in α- and β-glucosidase activities, and to decrease those involved in propionate production, especially in the HF subgroup. No treatment effect (*p* > 0.05) was found on pH, SCFAs, IgA, stool consistency and frequency.

**Conclusion:**

The tested resistant dextrin was effective in modulating the gut microbiota after four weeks of supplementation in healthy male volunteers, favoring the *Parabacteroides* genus and several related species (especially *P. distasonis*). The treatment effect was observed in both subgroups of dietary fiber intake, and was even more pronounced in the high-intake subgroup.

**Clinical trial registration:**

https://clinicaltrials.gov/, identifier NCT05105425.

## Introduction

Fibers are non-digestible carbohydrates, primarily polysaccharides composed of at least three monomers (1). They are key components of a healthy diet, although their intake is far lower than recommended in high-income countries (2, 3). Nutritional public health policies and medical advice most often blame modern chronic diseases, including obesity and associated cardiometabolic dysfunctions, on diets high in fat and sugar. Attention should be paid to low-fiber diets, which have also been linked to negative impacts on metabolic and cardiovascular functions, as well as gastrointestinal and mental health (4). The common denominator of these health impacts is likely to be the modulation of gastrointestinal microbiota. Indeed, the amount and type of fibers ingested via the diet is likely to shape the composition of the gut microbiota, to modify its function and to induce health effects (5).

The scarcity of fiber in the diet comes from insufficient consumption of fruit and vegetables (main suppliers of soluble fiber) and whole grains (main sources of insoluble fiber). Consuming fiber-enriched foods or dietary supplements can help compensate for this deficiency in those who are reluctant to change their diet. Soluble fibers such as resistant dextrins (RDs) can be easily incorporated into formulations because they have low viscosity and withstand many food processing conditions. RDs are glucose polymers derived from starch (6, 7). Their branched structure makes them resistant to hydrolysis by endogenous glucidolytic enzymes in the small intestine, and consequently available for bacterial fermentation (8). Supplementation in humans revealed that 87% to 98% of the supplemented RD is digested and fermented in the colon, as about 2–13% is excreted in the stool (9, 10).

The benefits of the RD used in the current study and its mechanism of action on gut microbiota have already been investigated through several clinical trials. First, the long-term gastrointestinal tolerance and the effects on colonic flora of two doses of RD (30 and 45 g) over 35 days were demonstrated in healthy male subjects (10). Both doses induced other beneficial effects, including the stimulation of lactobacilli growth, decrease in fecal pH and increase in fecal α- and β-glucosidase activities. At lower doses (8–20 g/day) given for 2 weeks, the supplementation favored saccharolytic bacteria (*Bacteroides*), while increasing β-glucosidase activity and reducing the abundance of the pathogen *Clostridium perfringens* (7). Utilizing advanced metagenomic sequencing techniques, a significant increase in the relative abundance of *Parabacteroides* and *Parabacteroides distasonis* was observed across three distinct clinical trials (11–13).

Several studies have further confirmed the health benefits of RD driven by microbiota shifts in blood glucose management, satiety, digestive health, and sustained energy release (14–16). Collectively, these results show that RD meets the ISAPP requirements for defining a prebiotic, i.e., “a substrate that is selectively utilized by host microorganisms conferring a health benefit” (17, 18), susceptible to claim for various health benefits.

Whether the impacts of the supplementation on gut microbiota depend on the level of dietary fiber intake remained to be investigated, and was the aim of the present study.

## Methods

### Study design and subjects

A randomized, double-blind, multicenter, placebo-controlled, parallel-arm trial was designed to investigate the effects of RD in healthy male adult volunteers with a low or high level of fiber intake. Two investigational centers (Biofortis clinical investigation units in Nantes and Paris, France) recruited participants. Recruitment and follow-up were performed between July 2021 and August 2023.

Potential participants were screened from a database of volunteers and examined during a selection visit (V0). Male volunteers fulfilling the following criteria were eligible to the study: age from 18 to 60 years, normal body mass index (BMI ≥ 18.5 and ≤ 25 kg/m^2^) and stable weight within the previous 3 months, normal intestinal transit during the run-in period (i.e., at most 3 stools/day and at least 5 stools/week), smoking fewer than 5 cigarettes per day, with stable food and physical activity habits during the previous 3 months, and agreeing to keep these habits unchanged throughout the study. During the run-in period (from V0 to V1), participants recorded their food intake during 4 days (three weekdays and one weekend day), using a food diary (Nutrilog^®^, Marans, France). The intake of dietary fibers was computed based on the reported meals and the Ciqual table of food nutritional composition, established and validated by the French food safety agency (ANSES). Subjects with a low level (≤15 g) or a high level (≥25 g) of mean daily fiber intake were eligible in the low fiber group (LF) or the high fiber group (HF), respectively. Female volunteers were not eligible to the study, to avoid fluctuations of the intestinal microbiota related to the hormonal cycle. Subjects with any chronic disease (including diabetes or gastrointestinal disease), under any treatment ended less than 4 weeks before inclusion that could affect the gut function, regular consumers of dietary supplements or products known to impact the intestinal microbiota, having followed a specific diet in the previous 3 months, or consuming more than 3 standard alcoholic drinks per day, were not eligible to the study.

This study was approved by an Independent Ethics Committee named *Comité de Protection des Personnes* (CPP) *NORD OUEST III* (Caen, France) and the French Health Authority (ANSM) was notified of this ethics approval. A signed informed consent form was acquired from each participant before randomization. The trial protocol was registered at ClinicalTrials.gov under NCT05105425.

### Randomization and blinding

Each subject was randomly assigned during the randomization visit (V1) to either the active product (RD) or a placebo according to the blocked randomization table generated using SAS^®^ software (version 9.4, SAS Institute Inc., Cary, NJ, United States).

The product allocation (ratio 1:1) used a dynamic randomization algorithm, designed to minimize imbalance between the two arms within the strata defined by the fiber intake (LF: ≤ 15 g/day/HF: ≥ 25 g/day) and center (Nantes / Paris). The investigator allocated the participants to a treatment arm through an online randomization interface, after confirmation of eligibility, thus minimizing the selection bias. Researchers and participants were blinded to intervention assignments until the database lock. RD and placebo were both formulated as powders and administered according to an identical dosing regimen. Packaging and labeling were indistinguishable between the two products.

### Intervention and procedures

**FIGURE 1:**
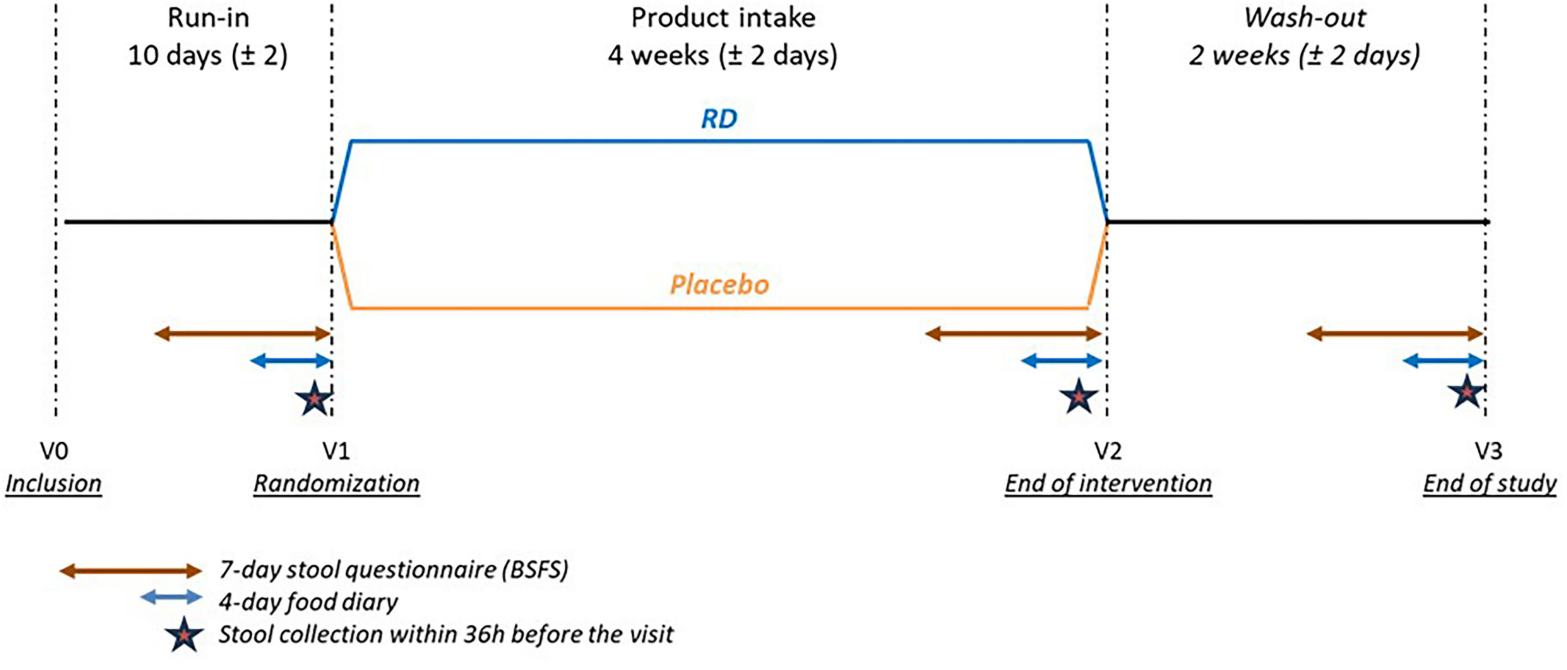
Study design. BSFS, Bristol Stool Form Scale; RD, resistant dextrin.

During the run-in period between the first visit (V0) and the randomization and treatment start visit (V1), all participants completed at home the Bristol stool form scale over a 7-day period, to assess the consistency and frequency of bowel movements. They also collected a stool sample at home within 36h before V1. The same steps were completed before the end-of-intervention visit (V2) and the end of study visit (V3), which was planned after two weeks of wash-out, following the end of supplementation (Figure 1). Dietary fiber intake was recorded before V2 and V3, and analyzed as detailed above.

During the 4-week intervention period, participants were instructed to consume orally one 15g-sachet of investigational product mixed with a beverage each morning. The product was either RD or placebo (NUTRIOSE FB 06^®^ or GLUCIDEX IT 21^®^, respectively; Roquette, France). NUTRIOSE^®^ FB06 is a RD, in other words, a glucose polymer derived from wheat starch. It is classified as a soluble dietary fiber with a total fiber content of about 85% and a mono- and disaccharide content ≤ 0.5% (6, 7). GLUCIDEX^®^ IT21 is a maize dried glucose syrup, completely digestible in the upper part of the gastrointestinal tract.

Participants were requested to maintain their regular diet and exercise routine and were instructed to refrain from taking any drug, food supplement, or other products that can directly impact the gut. Any adverse events or discomfort experienced by participants were to be reported to the investigator.

### Fecal parameters

Stool samples were collected at most 36h before V1, V2 and V3, to analyze microbiota composition, function and diversity, and to measure fecal pH, short chain fatty acid (SCFA) level and secretory immunoglobulin A (IgA) level. Stools were collected at home and kept between 2 and 8°C in a cooler until returned to the investigator at the next visit. They were then aliquoted and stored at −80°C for further analysis, centralized at the Biofortis laboratory (Nantes, France).

Microbiota was analyzed by shotgun metagenomics. Briefly, DNA was extracted from stool samples, sequenced by next generation sequencing (MGI DNBSEQ-G400). The targeted metagenomic sequences were analyzed using an in-house bioinformatics pipeline, following several steps starting with host sequences deletion [using Bowtie2 v2.4.5 (19) and quality-filtering (using Fastp v0.23.2 (20)]. Then, the filtered reads were aligned against a reference database using Kraken2 v2.1.2 (21) to describe the community profiles for bacteria, DNA viruses and fungi. Functional annotation was performed using HUMAnN3 for gene ontology (GO) that describes cellular component, molecular function and biological process (22); enzyme commission (EC), which is a 4-level classification of enzymes (23); and MetaCyc pathways, that describes the pathways involved in both the primary and secondary metabolism, as well as associated metabolites, reactions, enzymes, and genes (24).

Fermentative activity of the fecal microbiota was assessed by measuring fecal SCFA concentrations (acetate, propionate, butyrate) using gas chromatography coupled with mass spectrometry (25). A pH-meter equipped with a matrix-specific probe (pHenomenal SPEAR 220 probe on pHmeter 1000 L; VWR) was used to measure fecal pH. Secretory IgA assay in stool was performed using an enzyme-linked immunosorbent assay (ELISA kit ref RIC6100R; BioVendor).

### Outcomes

The primary endpoint of the study was the relative abundance of the *Parabacteroides* genus in intestinal microbiota at the end of supplementation (V2). This parameter was also assessed at the end of the wash-out (V3) as a secondary endpoint. The relative abundances of other bacterial taxa were also explored: *Parabacteroides* species, *Bacteroides* genus, *Bacillota* phylum (formerly named *Firmicutes*), species associated to *Clostridium Clusters IV* and *XIVa* (26) (see Supplementary Table 1 for details). The abundance of bacterial genes associated to α- and β-glucosidase activity and to SCFA metabolism were also studied at the end of supplementation (see Supplementary Table 2 for details). Finally, the fecal microbiota was analyzed for its functional and taxonomic α-diversity (observed richness, Shannon and Inverse Simpson indexes) and β-diversity (Bray-Curtis and Jaccard dissimilarity indexes).

### Statistical analysis

In this pilot study, no formal sample size calculation was performed, but the number of participants was derived from various guidelines suggesting 10–40 subjects per group of interest. It was planned to include 30 subjects per treatment arm (RD and placebo) and subgroup of fiber intake (HF and LF), for a total of 120 participants. For all statistical tests, the 0.05 level of significance was used to declare a statistically significant effect.

Statistical analyses related to microbiota were performed using R software version 4.3.3 (R Core Team, Vienna, Austria), while other analyses were performed using SAS software version 9.4 on the intention-to treat population (ITT; all randomized participants), and per-protocol population (PP; participants from the ITT without any major protocol deviations) as supportive analyses.

The primary endpoint was analyzed using a generalized linear mixed model (GLMM) with fixed effects: treatment (RD or placebo), visit (V2 or V3), group of fiber intake (HF or LF), 2nd and 3rd order interactions between these effects, and baseline value (at V1), and a random effect on subject:


Y=t⁢r⁢e⁢a⁢t⁢m⁢e⁢n⁢t+v⁢i⁢s⁢i⁢t+g⁢r⁢o⁢u⁢p+t⁢r⁢e⁢a⁢t⁢m⁢e⁢n⁢t*v⁢i⁢s⁢i⁢t

   +t⁢r⁢e⁢a⁢t⁢m⁢e⁢n⁢t*g⁢r⁢o⁢u⁢p+v⁢i⁢s⁢i⁢t*g⁢r⁢o⁢u⁢p+t⁢r⁢e⁢a⁢t⁢m⁢e⁢n⁢t

$$\;\;*visit*group+Y_{V\,1}+subject_{r\,a\,n\,d\,o\,m}$$

The random intercept on center was removed after failing to converge for most of the tested endpoints. The model was applied on log-transformed data. The tested alternative hypothesis was a higher relative abundance of *Parabacteroides* at V2 with RD than with placebo (i.e., superiority).

Distributional assumptions of the primary GLMM were assessed by visual inspection of residual plots. Missing data at V2 and V3 were handled naturally by likelihood-based estimation in the GLMM framework. The implicit missing at random assumption was deemed reasonable in this setting, due to the small fraction of missing samples.

Furthermore, a Wilcoxon rank sum test, which does not adjust for any other factor/covariate, was performed on log-transformed data to generate illustrations. It also served as a nonparametric fallback when assumptions from parametric models were not met.

The assessment of microbiota components using differential abundance analysis (DAA) between compared arms was performed *post-hoc* using a set of univariate statistical models, which capture different aspects of the data, in line with the recommendation from recent benchmarking studies (27, 28), that found that no single statistical method is uniformly better for all scenarios. Three statistical models were considered, achieving a good trade-off between detection power and rate of false discoveries: Maaslin2 (29), ALDEx2 (30) and ANCOM-BC (31). Given the high-dimensional nature of DAA (hundreds of taxa tested simultaneously), individual residual diagnostics were not performed, consistent with standard practice in microbiome studies. No imputation method was used to replace missing data.

Another *post-hoc* analysis was conducted to further explore the microbiome profiles of participants who were particularly responsive to RD supplementation. Responders were defined as subjects whose relative abundance in *Parabacteroides* at least doubled from V1 to V2. Statistical comparisons of responder rates were performed using Fisher’s exact tests.

All comparisons between the two products on gut microbiota characteristics, as well as on fecal pH, fecal SCFA, IgA and average stool consistency (BSFS) were analyzed using the same GLMM as for the primary endpoint (albeit applied in log-scale for abundance of microbiota components), using the same methods to check normality and handle missing data. For each participant, the dissimilarity of microbiota between V1 and V2, and between V1 and V3 was calculated; the dissimilarity was then analyzed using the same model excluding the value at V1. A mixed Poisson regression model with the same fixed effects and random effect as for analysis of the primary endpoint was used to analyze the daily number of bowel movements over the 7-day period.

## Results

### Characteristics of participants

**FIGURE 2:**
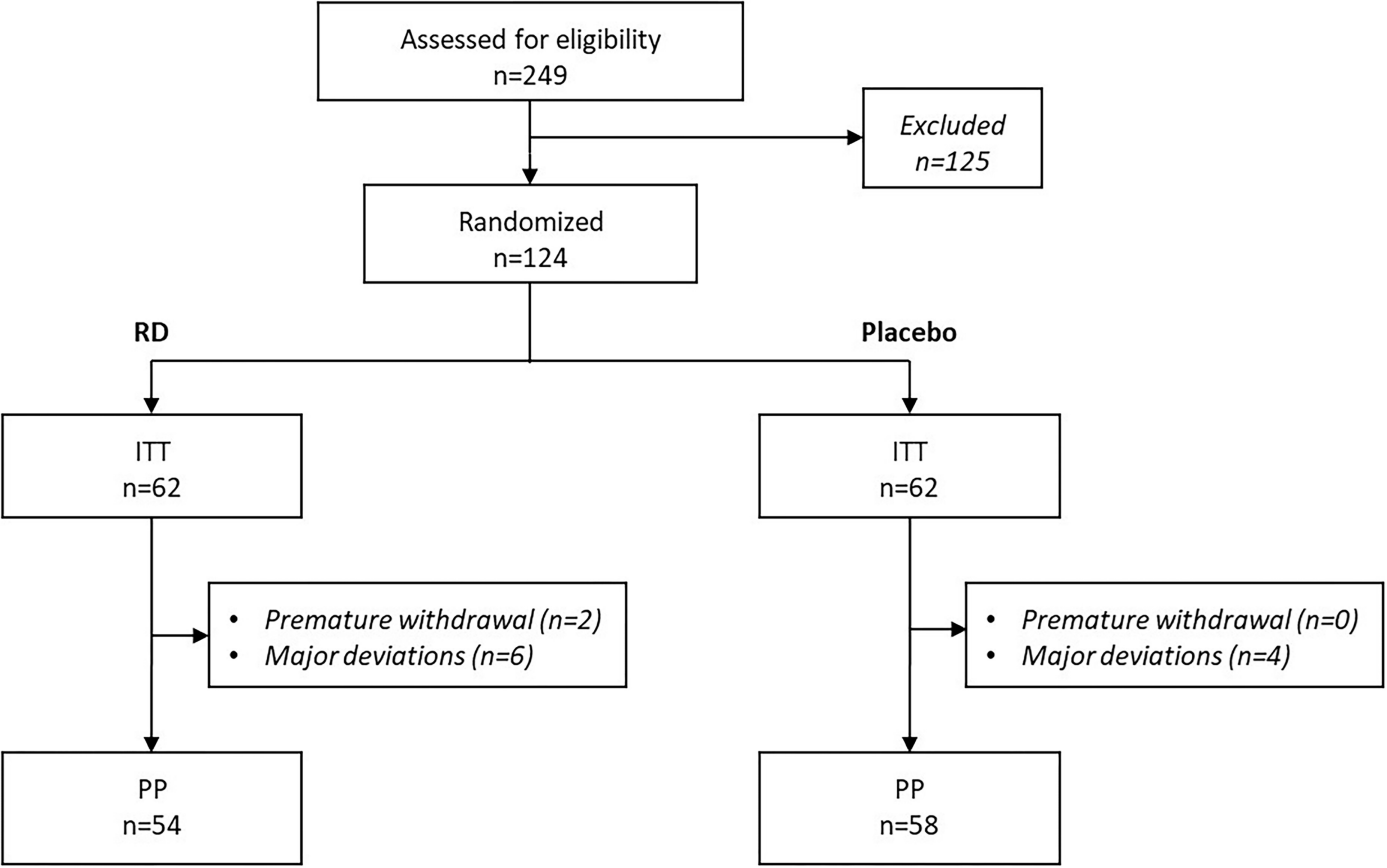
Study flow chart. ITT, intention to treat population; PP, per protocol population; RD: resistant dextrin.

A total of 249 subjects were screened in this study, of whom 124 subjects (49.8%) were randomized and formed the ITT population (62 in the RD arm, 62 in the placebo arm). This included 67 individuals in the HF subgroup and 57 in the LF subgroup, according to their mean daily fiber intake at baseline (≥25 g and ≤ 15 g, respectively). Overall, 12 participants of the ITT population (9.7%) presented major deviations to the protocol and were therefore excluded from the PP population (*N* = 112). Indeed, 2 participants prematurely stopped the study before the end of treatment (V2) and 10 participants finished the study but were excluded primarily due to a deviation on the inclusion criterion on normal intestinal transit (Figure 2).

Main baseline parameters of the ITT population by treatment arm are presented in Table 1. In the whole cohort, the mean age of participants was 36.2 ± 11.6 years, with a mean BMI of 22.8 ± 1.7 kg/m^2^ corresponding to a normal weight. Data were quite similar between randomization arms. The mean dietary fiber intake was close to 31 g/day in the HF subgroup and 12 g/day in the LF subgroup, regardless of the treatment arm. The baseline relative abundance of *Parabacteroides* was quite similar among arms and subgroups, ranging from 1.1 to 1.7%. The average stool frequency (1.38 ± 0.52 stools/day) and stool consistency (3.72 ± 0.70 a.u. on the BSFS) was similar at baseline in the HF and LF subgroups, whatever the treatment arm, and corresponded to a normal transit with an intermediate stool consistency. No meaningful difference was observed between the ITT and PP populations at baseline, nor between arms.

**TABLE 1:** Descriptive baseline characteristics (at V1) per study arm and fiber group in the ITT population.

Variable	RD			PLACEBO		
	HF + LF (*n* = 62)	HF (*n* = 33)	LF (*n* = 29)	HF + LF (*n* = 62)	HF (*n* = 34)	LF (*n* = 28)
Age (years)						
Mean (SD)	35.9 (11.5)	37.4 (11.4)	34.3 (11.6)	36.5 (11.8)	38.9 (12.2)	33.5 (10.8)
(Min; Max)	(18.0; 58.0)	(18.0; 58.0)	(20.0; 57.0)	(19.0; 59.0)	(19.0; 57.0)	(20.0; 59.0)
Median (Q1; Q3)	35.5 (27.0; 44.0)	38.0 (31.0; 45.0)	31.0 (26.0; 42.0)	34.0 (27.0; 47.0)	41.0 (29.0; 49.0)	30.0 (26.0; 39.5)
BMI (kg/m2)						
Mean (SD)	23.0 (1.9)	23.1 (2.0)	22.8 (1.7)	22.6 (1.6)	22.6 (1.6)	22.7 (1.7)
(Min; Max)	(18.9; 26.6)	(18.9; 26.6)	(19.7; 25.5)	(19.0; 26.6)	(19.3; 25.1)	(19.0; 26.6)
Median (Q1; Q3)	23.1 (21.6; 24.5)	23.4 (22.1; 24.5)	22.6 (21.6; 24.4)	22.8 (21.7; 23.9)	22.8 (21.2; 24.1)	23.0 (21.8; 23.8)
Daily fiber consumption (g/day)						
Mean (SD)	22.3 (11.5)	31.0 (8.8)	12.3 (2.4)	22.4 (11.4)	30.7 (8.9)	12.4 (2.1)
(Min; Max)	(5.7; 67.4)	(25.1; 67.4)	(5.7; 15.2)	(6.5; 67.1)	(25.0; 67.1)	(6.5; 14.8)
Median (Q1; Q3)	25.1 (12.6; 28.4)	27.7 (26.1; 30.4)	12.5 (11.0; 14.6)	25.2 (13.0; 28.5)	27.8 (25.6; 30.8)	12.8 (11.4; 14.0)
Relative abundance of Parabacteroides (%)						
Mean (SD)	1.4 (1.7)	1.1 (1.5)	1.7 (1.9)	1.4 (1.2)	1.4 (1.3)	1.3 (1.1)
(Min; Max)	(0.1; 9.2)	(0.1; 8.6)	(0.1; 9.2)	(0.1; 5.8)	(0.1; 5.8)	(0.1; 3.6)
Median (Q1; Q3)	0.8 (0.4; 1.7)	0.8 (0.3; 1.4)	0.9 (0.5; 2.6)	1.1 (0.6; 1. 7)	1.1 (0.7; 1.6)	1.0 (0. 4; 1.9)
Average stool frequency (number of stools/day)						
Mean (SD)	1.38 (0.48)	1.46 (0.56)	1.29 (0.38)	1.38 (0.56)	1.39 (0.58)	1.37 (0.55)
(Min; Max)	(0.57; 2.71)	(0.57; 2.71)	(0.57; 2.43)	(0.57; 3.29)	(0.71; 3.00)	(0.57; 3.29)
Median (Q1; Q3)	1.29 (1.00; 1.57)	1.29 (1.00; 1.86)	1.29 (1.00; 1.43)	1.14 (1.00; 1.57)	1.14 (1.00; 1.57)	1.21 (1.00; 1.64)
Average stool consistency (arbitrary unit)						
Mean (SD)	3.69 (0.77)	3.70 (0.87)	3.68 (0.64)	3.76 (0.64)	3.76 (0.62)	3.76 (0.68)
(Min; Max)	(1.33; 6.00)	(1.33; 5.14)	(2.71; 6.00)	(2.00; 5.10)	(2.00; 4.67)	(2.50; 5.10)
Median (Q1; Q3)	3.71 (3.15; 4.22)	3.83 (3.09; 4.27)	3.63 (3.17; 4.00)	3.72 (3.43; 4.18)	3.89 (3.50; 4.13)	3.61 (3.31; 4.40)

ITT, intention to treat population; HF, high dietary fiber group; LF, low dietary fiber group; HF+LF, both groups. RD, resistant dextrin. Stool frequency and stool consistency were recorded for 7 days before the visit on a diary, using the Bristol stool form scale ranking stools from 1 (separate hard lumps) to 7 (watery).

Compliance to the supplementation was high no matter the treatment arm and the visit (ratio of effective / theoretical sachets intake > 97%). The mean dietary fiber intake decreased at V2 and V3 in the HF subgroups, as compared to baseline, while increasing in the LF subgroups (Supplementary Table 3).

### Gut microbiota composition

#### *Parabacteroides* is stimulated by the RD supplementation

The *Parabacteroides* genus was retrieved in all fecal samples, regardless of visit, treatment and fiber intake. The mean relative abundance at baseline was similar in both arms (1.4%) and comparable in the two subgroups of fiber intake (LF: 1.5%; HF: 1.3%) (see Supplementary Table 4). It increased to 5.5% (95%CI: [4.8; 6.1]) at V2 with the RD supplementation but remained stable with the placebo (1.4% [0.8; 2.1]). This difference was statistically significant (*p* < 0.0001), according to the linear mixed model. Two weeks later, at the end of the wash-out, the relative abundance returned to 1.2% [0.5; 1.8] in the RD arm and 1.3% [0.8; 2.1] in the placebo arm (*p* = 0.5129).

**FIGURE 3:**
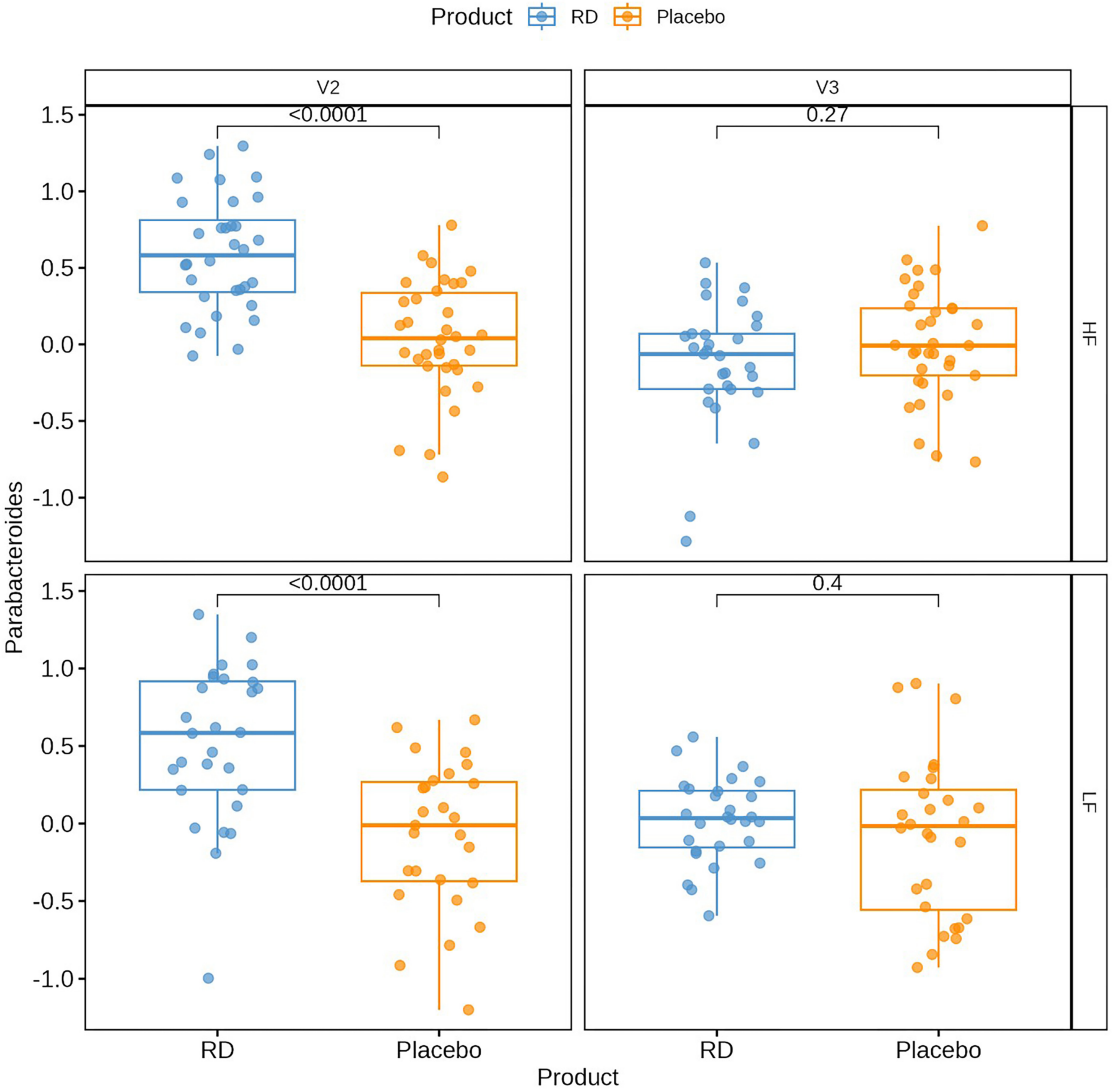
Relative abundance of the *Parabacteroides* genus in high (HF) and low dietary fiber (LF) groups. Plots show the log-transformed relative abundance of the *Parabacteroides* genus in stool samples [log(%)] at V2 (end of supplementation) and V3 (end of study). *P*-values are from a Wilcoxon rank sum test performed on log-transformed data. Data are for the ITT population (*N* = 124; missing: 3 at V2 and 6 at V3). HF: high dietary fiber group; LF: low dietary fiber group; RD: resistant dextrin.

A significant treatment effect was also found in the two subgroups at V2 (5.6% [4.7: 6.5] vs. 1.5% [0.6; 2.4], p < 0.0001 in HF; 5.3% [4.3; 6.2] vs. 1.4% [0.4; 2.3] in LF), but not at V3 (1.4% [0.4; 2.3] vs. 1.4% [0.5; 2.2], *p* = 0.94 in HF; 1.0% [0.1; 2.0] vs. 1.6% [0.7; 2.6] in LF, *p* = 0.41). Figure 3 illustrates the distribution of data per treatment arm and fiber group at V2 and V3, together with p-values from the Wilcoxon rank sum test, which confirmed that the relative abundance of *Parabacteroides* was significantly higher in the RD arm at V2, both in the HF and LF subgroups.

#### The RD supplementation significantly impacts several species from the *Parabacteroides* genus and *Bacillota* phylum, more often in HF than in LF subjects

The significant effect of RD intake on the entire *Parabacteroides* genus was also observed at the level of various children species (Table 2). In particular, *P. distasonis* showed the highest abundance at V2 in the RD arm (2.6 ± 2.8%) as compared to placebo (0.5 ± 0.5%). Overall, the *Bacillota* phylum was almost significantly less abundant at the end of supplementation in the RD arm than in the placebo arm (observed mean: 55.4 ± 11.7% vs. 59.0 ± 13.3%; *p* = 0.0545). However, a higher relative abundance was found for several species, including species from the *Clostridium* genus, while it was lower for other species (Table 2). Most of the *Bacillota* species were part of *Clostridium* cluster IV and *Clostridium* cluster XIVa. However, no statistically significant between-arm differences were observed for these clusters. At V2, the relative abundance of *Clostridium cluster IV* was 9.0 ± 4.1% in the RD arm and 8.9 ± 4.6% in the placebo arm; it was 7.6 ± 4.2% and 8.2 ± 4.1% for *Clostridium cluster XIVa.*

**TABLE 2:** Between-arm difference in the relative abundance of bacterial taxa at the end of supplementation (V2), in high (HF) and low (LF) dietary fiber subgroups and in the whole cohort (HF+LF) in the ITT population.

Phylum	Genus	Species	HF+LF	HF	LF
*Bacteroidota*	*Parabacteroides*	*All*	**↗ (*p* < 0.0001)**	**↗ (*p* < 0.0001)**	**↗ (*p* < 0.0001)**
		*distasonis*	**↗ (*p* < 0.0001)**	**↗ (*p* < 0.0001)**	**↗ (*p* < 0.0001)**
		*merdae*	↗ (*p* = 0.0886)	↗ (*p* = 0.0516)	NS
		*goldsteinii*	↗ (*p* = 0.0727)	NS	NS
		*CT06*	**↗ (*p* = 0.0348)**	↗ (*p* = 0.072)	NS
		*Unclassified*	**↗ (*p* < 0.0001)**	**↗ (*p* < 0.0001)**	**↗ (*p* < 0.0001)**
*Bacillota*	*All*	*All*	↘ (*p* = 0.0545)	NS	↘ (*p* = 0.0825)
	*Clostridium*	leptum*	**↗ (*p* = 0.037)**	**↗ (*p* = 0.0163)**	↗ (*p* = 0.0802)
		sporosphaeroides*	**↗ (*p* = 0.0013)**	**↗ (*p* = 0.0061)**	↗ (*p* = 0.0582)
		symbiosum**	↗ (*p* = 0.0592)	NS	NS
		aminophilum**	**↗ (*p* = 0.0008)**	**↗ (*p* = 0.0013)**	NS
	*Eubacterium*	cellulosolvens**	**↗ (*p* = 0.0002)**	**↗ (*p* = 0.0003)**	↗ (*p* = 0.0935)
	*Lacrimispora*	sphenoides**	↗ (*p* = 0.0920)	↗ (*p* = 0.0687)	NS
		aerotolerans**	**↗ (*p* = 0.0029)**	**↗ (*p* = 0.0178)**	↗ (*p* = 0.0619)
	*Ruminococcus*	torques**	**↘ (*p* = 0.0049)**	**↘ (*p* = 0.0183)**	↘ (*p* = 0.0992)
		lactaris**	**↘ (*p* = 0.0453)**	NS	NS
	*Mediterraneibacter*	faecis**	**↘ (*p* = 0.0041)**	**↘ (*p* = 0.0203)**	↘ (*p* = 0.0781)

Only taxa with at least one statistically significant difference (*p* < 0.05, bold) or tendency to difference (*p* < 0.1) between the resistant dextrin (RD) and placebo arms are presented, as per the generalized linear mixed model applied to log-transformed data. ↗ (blue) and ↘ (orange): higher and lower relative abundance with RD, respectively, as compared to the placebo; HF, high dietary fiber group; LF, low dietary fiber group; NS, not significant;

* members of Clostridium cluster IV; ** members of Clostridium cluster XIVa. Data are for the ITT population at V2 (*N* = 124, 3 missing data).

While these differences were observed in the entire population, most of them were found in the HF subgroup as well, but to a lesser extent in the LF subgroup. In addition, all the changes in microbiota composition were temporary, as they were observed at the end of the 4-week supplementation (V2) but were no longer observed 2 weeks later (V3). Of note, the *Bacteroides* genus, which did not present any difference in relative abundance at V2 between arms, was significantly more abundant at V3 in the RD arm (*p* = 0.0355), and particularly in the HF subgroup (*p* = 0.0184).

**FIGURE 4:**
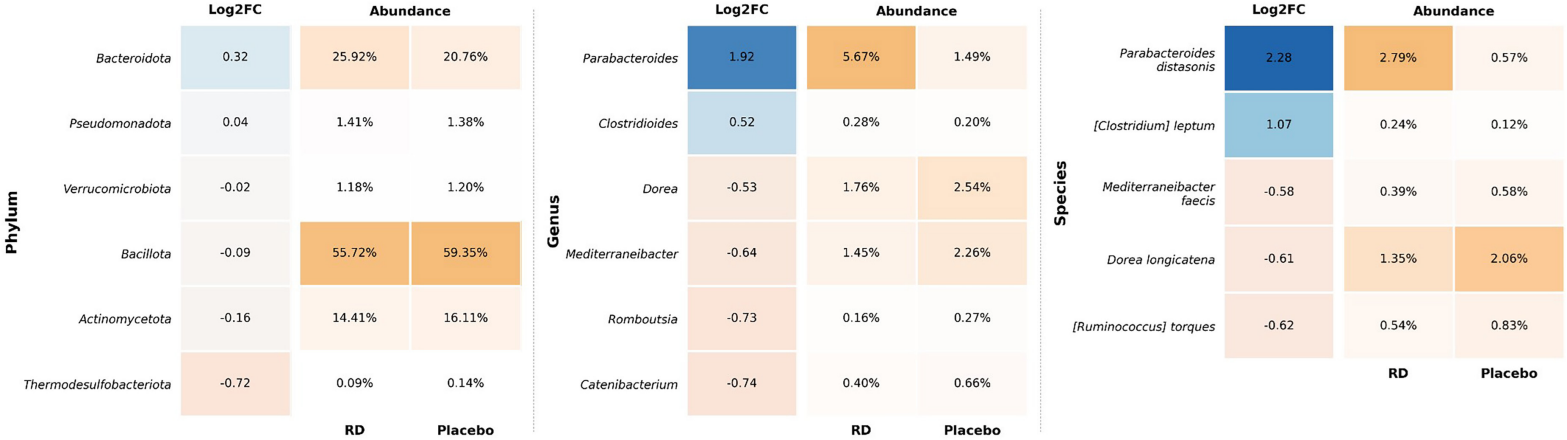
Differential analysis of abundance in fecal microbiota between the RD and placebo arms at the phylum, genus and species levels, at the end of supplementation (V2). Only taxa with significantly different relative abundance between groups are presented, as per three statistical models: ALDEx2 (30) and/or MaasLin2 (29), and/or ANCOM-BC (31). Log_2_FC: Log_2_ of the mean fold change, as computed from sample statistics. This value indicates how much the relative abundance of the taxon differs between groups (the value is reported on a logarithmic scale to base 2). Only phyla, species and genera present in almost all samples are presented (prevalence ≥ 95%, regardless of the group RD/placebo). The intensity of the orange color in the abundance panels increases with the relative abundance, with scales defined independently for each taxonomic level. For Log2FC, blue indicates taxa that are relatively more abundant in the RD arm, whereas orange indicates taxa enriched in the placebo arm. Color intensity increases with the absolute log2 difference in mean abundance between arms. Data are for the ITT population (*N* = 124, 3 missing). HF, high dietary fiber group; LF, low dietary fiber group; RD, resistant dextrin.

The effects of the supplementation on the composition of the microbiota were also analyzed by a DAA at the phylum, genus and species levels (Figure 4). This analysis confirmed the results of the generalized mixed linear model on the whole cohort (HF+LF). The largest differences in relative abundance between the RD and placebo arms were observed for the *Bacteroidota* phylum (25.92% vs. 20.76%), the *Parabacteroides* genus (5.67% vs. 1.49%) and *P. distasonis* (2.79% vs. 0.57%). The same analysis performed at V3 did not show any significant difference between the supplementation groups, except for *Bifidobacterium pseudocatenulatum*, which was more abundant in the RD group.

**FIGURE 5:**
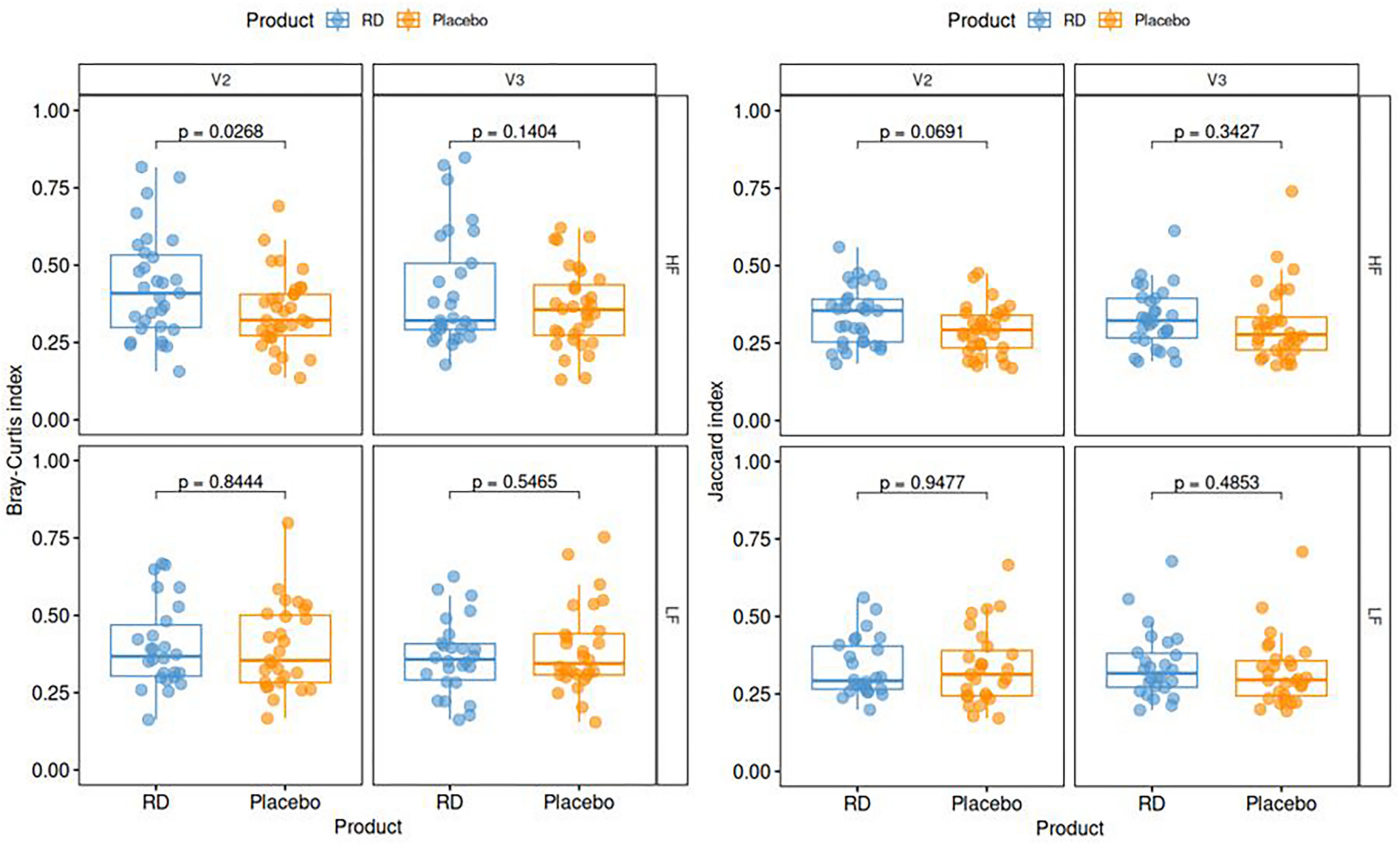
Beta-diversity indexes per treatment arm and dietary fiber group. Plots show dissimilarity of fecal microbiota between baseline and V2 (end of supplementation) or V3 (end of study), as per the Bray-Curtis index (left) and the Jaccard index (right). *P*-values correspond to the inter-arm difference tested in a generalized mixed model. Data are for the ITT population (*N* = 124; missing: 3 at V2 and 6 at V3). HF, high dietary fiber group; LF, low dietary fiber group; RD, resistant dextrin.

No significant treatment effect was observed regarding the α-diversity indexes of bacterial taxa (observed richness, Shannon and Inverse Simpson), whether at the end of supplementation (V2) or at the end of the wash-out (V3), meaning that the distributions (richness and evenness) of the different species were comparable. Regarding β-diversity (Figure 5), i.e., the measure of dissimilarity between different timepoints, we observed a statistically significant difference between RD and placebo at V2 in the HF subgroup, with the Bray-Curtis index (*p* = 0.0268). Based on both presence/absence and relative abundances of the species, it was significantly higher in the active group, meaning that the evolution of the bacterial ecosystem was reshaped by RD intake, but only in subjects who already had a fiber-rich diet. Despite a trend observed in the same subgroup, the difference was not statistically significant for the Jaccard index, based on the presence/absence of the species.

#### The RD supplementation benefits to taxa with α– and β-glucosidase activity

The fecal microbiota was also analyzed from a functional perspective. The relative abundance of bacterial genes associated with α-glucosidase activity (EC 3.2.1.20) was significantly higher after RD supplementation relative to the placebo, but only in the HF subgroup (*p* = 0.0020); such difference was also found after the wash-out in the LF subgroup (*p* = 0.0385) and the HF subgroup (although almost significant: *p* = 0.0511). In addition, significant differences between the HF and LF subgroups were found at V2 (*p* = 0.0013) and V3 (*p* = 0.0042), meaning that the effect of a 4-week consumption of RD on relative abundance of bacterial genes associated with α-glucosidase activity is dependent on subjects’ fiber intake. Similar results were found for genes associated with β-glucosidase activity (EC 3.2.1.21), which were more abundant after the RD supplementation in the whole population (*p* = 0.0023) and in the HF subgroup (*p* = 0.0019).

Regarding SCFAs, the relative abundance of genes involved in the metabolism of propionate (EC 2.8.3.1 and EC 2.7.2.15) was significantly lower in the RD arm than in the placebo arm at V2 in the HF subgroup (*p* = 0.006); the difference was almost significant in the whole population (*p* = 0.0673). No difference was found regarding genes associated with butyrate metabolism (EC 2.7.2.7 and EC 2.8.3.8) nor with acetate metabolism (EC 2.7.2.1, EC 2.8.3.8 and EC 2.8.3.10), except for EC 2.8.3.12, which was less abundant after RD supplementation in LF subjects only (*p* = 0.0346). Two weeks after the end of supplementation (V3), no statistically significant differences in relative abundance were observed between the treatment arms.

The overall gene composition was investigated from a broader perspective through α and β diversity indexes, but no statistically significant differences were found between treatment arms in the ITT population, meaning that the variation of genes observed after 4 weeks of supplementation was equivalent between the two treatment arms.

#### Response to the RD supplementation is higher in subjects with a high-fiber diet

*Post-hoc* analyses explored the status of responder / non-responder (R / NR), based on doubling of the relative abundance of *Parabacteroides* from V1 to V2. In the entire population, 74.6% of subjects were R in the RD arm, vs. 16.4% in the placebo arm (p < 0.001). The proportion of R after the RD supplementation was higher in the HF subgroup than in the LF subgroup (81.3% vs. 66.7%), although not significantly (*p* = 0.2399), while it was comparable in the placebo arm (17.9% in HF vs. 15.2% in LF; *p* = 1).

#### The RD supplementation does not induce meaningful effects on other fecal parameters

When comparing SCFAs in fecal samples from the RD vs. placebo arm, we only observed a significantly lower level of butyrate at V2 in the overall population (mean estimates: 15.2 vs. 19.3 μmol/g; *p* = 0.0113) and in the LF subgroup (14.3 vs. 19.6 μmol/g; *p* = 0.0273).

Finally, no statistically significant differences were observed between RD and placebo for fecal pH, fecal IgAs and stool frequency. We only observed a trend toward a higher stool frequency and more consistent stools after RD than placebo (see Supplementary Table 5 for details).

Correlation analyses between SCFA concentrations, pH, and secretory IgA levels on the one hand, and Responder/Non-Responder status on the other, show no significant results, whether at baseline or at the end of supplementation.

### Tolerance

As previously mentioned, the impact of the active treatment on the gastrointestinal transit was minor. Some treatment-emergent adverse events were recorded, but all were of mild or moderate intensity, and none yielded any action on product intake. Therefore, the prebiotic was very well tolerated and no safety concern related to RD intake (15 g/day for 4 weeks) was raised during the study.

## Discussion

There is limited research assessing the impact of dietary supplements across high- and low-fiber diets. The present study successfully compared the effects of RD vs. placebo in two groups of participants well differentiated by their fiber intake, and pointed out different effects related to this characteristic.

Beyond the subgroup analysis, the effects of supplementation with the prebiotic fiber NUTRIOSE^®^ on the entire cohort were consistent with findings from previous clinical trials, showing its ability to induce changes in parameters related to intestinal microbiota. RD supplementation leads, among others, to an increase in relative abundance of *P. distasonis* (12, 13), an increase in the proliferation of saccharolytic bacteria (*Bacteroides*, *Parabacteroides*), and an increase in potential α- and β-glucosidase activity (7, 9). The current study confirms the ability of RD to boost the growth of the *Parabacteroides* genus within gut microbiota, and especially that of *P. distasonis*, in healthy adult male volunteers. Consistently, the supplementation also resulted in strengthening the saccharolytic potential of gut microbiota, as observed through the increased relative abundance of bacterial genes involved in α- and β-glucosidase activities, present in *Parabacteroides*.

These findings indicate that the investigated RD differs from other dietary fibers in its capacity to be a substrate for specific species. Supplementation with RD is not just about increasing the global amount of fiber ingested, it provides a specific quality of fiber that results in modifying the microbiota from a taxonomic and functional perspective. This effect on *Parabacteroides* appears transient as it was no longer found 2 weeks after the end of supplementation. However, some prolonged effects of the active intervention were observed 2 weeks after it ended: a higher relative abundance of the *Bacteroides* phylum, and of the *Bifidobacterium pseudocatenulatum* species, particularly in the group with a high-fiber diet, as compared to placebo. In animal studies, this species has been linked to the reduction of obesity-associated inflammation, suggesting potential long-term beneficial effects (32). Furthermore, it cannot be excluded that a longer supplementation would have yielded longer effects on the gut microbiota.

The specificity of the current study was the inclusion of participants with high and low levels of dietary fiber intake. At baseline, this nutritional difference did not translate into differences in the relative abundance of *Parabacteroides* in their fecal microbiota. Both subgroups benefited from the prebiotic supplementation with regard to the growth of *Parabacteroides* and species of interest such as *P. distasonis* and several *Clostridium* species. Even further, the effect of RD was found to be stronger in participants who were used to consuming a high-fiber diet, as compared to a low-fiber diet, regarding: (1) the number of taxa whose abundance was significantly modified by the treatment, and (2) the proportion of responders (i.e., with abundance of *Parabacteroides* at least doubled). Similarly, the response to an inulin-type fructan prebiotic was also found to be dependent on the dietary fiber intake in a cross-over trial: high-fiber consumers gained a greater benefit from the intervention (33). Beyond differences related to the prebiotic and resulting microbial changes, these two studies highlight the importance of assessing dietary fiber intake in nutrition clinical trials, in line with the recommendations of a recent perspective article (34).

How an individual responds to a dietary intervention is hardly predictable, as the response relies on the initial composition and diversity of the microbiota, but also on the host phenotype. Nevertheless, the percentage of responders to RD, defined as doubling of *Parabacteroides* relative abundance, is quite high compared to other soluble fibers (35).

*Parabacteroides* exhibits physiological traits related to metabolizing carbohydrates and producing SCFAs (36). Some *Parabacteroides* species may be considered promising candidates for next-generation probiotics because of their potential health benefits (37). Indeed, according to *in vitro* or pre-clinical data, *P. distasonis* exhibits anti-inflammatory and epithelial barrier reinforcing properties (38). Moreover, *P. distasonis* produces succinate (39), a precursor of propionate (40), which has been shown to increase with RD supplementation in *ex-vivo* experiments (41). Data on *P. goldsteinii* suggested positive effects on obesity (25, 36, 42). The findings are slightly more controversial for *P. merdae*. Indeed, it was frequently found in the gut of hypertensive patients (43) and exhibited higher abundance in individuals with polycystic ovary syndrome (44). In contrast, some strains, such as *P. merdae* AS106, induce the release of anti-inflammatory cytokines in immune cell cultures (25). Specific strains could thus be good probiotic candidates.

*Many* species within the *Clostridium* Clusters IV and XIVa are recognized as producers of SCFAs, known as a major source of energy for the colonocytes. This is notably observed in species such as *C. leptum* and *C. sporosphaeroides* (from Cluster IV), as well as C. *aminophilum*, *C. symbiosum*, *L. sphenoides*, *L. aerotolerans*, and *E. cellulosolvens* (from Cluster XIVa) (45–47). Additionally, various other beneficial characteristics including anti-inflammatory properties have been associated with these species. Hence, they are also considered promising probiotic candidates (45). We observed an increase in relative abundance of these species, supporting the prebiotic nature of RD. However, these changes were not associated with increased levels of SCFAs in fecal samples. Furthermore, we found lower levels of butyrate in the active arm as compared to the placebo arm after a 4-week supplementation, which is not consistent with previous data on RD (13). In addition, we did not observe any product effect on the abundance of genes involved in butyrate production. It must be noted that the analysis was performed on a fixed quantity of stool, not proportional to the total quantity of stool excreted, and without considering the inter-sample difference in fecal water content. Besides, the concentration of SCFAs in the stool might not accurately reflect their overall production. Indeed, the level of SCFAs found in fecal samples results from the amount produced by the microbiota minus the amount absorbed through or used by the epithelium in the large intestine (10, 48). Therefore, the biological relevance of fecal SCFA concentrations as sole readouts of RD-induced microbial changes remains limited. More integrative and quantitative approaches would be required to fully assess the functional consequences of these microbiota shifts.

Conversely, we observed a decrease in relative abundance of species belonging to the *Ruminococcus* genus after 4 weeks of supplementation, especially *R. torques*, *R. lactaris*, along with *M. faecis* (previously known as *R. faecis* before a recent reclassification). The reduction in *R. torques* is notable given its role as a mucolytic bacterium, which has been implicated in the pathogenesis of inflammatory bowel disease (49). This suggests that the observed changes in microbial composition could positively influence gut health and mucosal integrity.

The opposite variations of taxa observed in this study can be explained by the competitive exclusion principle (50). Indeed, *Parabacteroides*, *Clostridium*, *Eubacterium* and *Ruminococcus* are fibrolytic bacteria, which compete for fiber fermentation and thus cannot maintain a stable coexistence. Therefore, taxa with even a slight advantage proliferate at the expense of others. It can be assumed that this ecological advantage relies on different sets of enzymes and metabolic pathways to achieve fiber fermentation.

We did not observe any significant differences between treatment arms in terms of α-diversity indexes, indicating that none of the previously described changes impact the general structure of the microbiome. Regarding β-diversity, statistically significant differences were observed with the Bray-Curtis index (which combines quantitative size and shape of the microbial community), but not with the Jaccard index (which depicts qualitative dissimilarity only). These results suggest that the variation of intestinal microbiota observed after 4 weeks of RD supplementation is more likely linked to the relative variation of dominant taxa than to strong modifications in microbiota composition. This increased β-diversity was only observed in high fiber consumers, as it was for α- and β-glucosidase related genes as well. This is consistent with observations made on the taxonomic composition.

Two weeks after the end of supplementation, we observed an increase in relative abundance of the *Bacteroides* genus, concomitant to the decrease of *Parabacteroides* and *Clostridium*. This evolution is likely to correspond to an ecological succession pattern, commonly observed after an intervention on a balanced microbiota.

This study presents some limitations. First, as participants were all male with a normal BMI, further research is needed to confirm applicability to the general population, and particularly to women. Second, subjects were selected in subgroups based on their usual fiber intake, assessed using a self-completed 4-day food questionnaire. This method was chosen for its practicality, and although it is not the most accurate, the fiber intake thresholds used to define subgroups were extreme, thus the questionnaire was able to discriminate them properly. Moreover, we did not consider the type of fiber ingested, which may shape the microbiota and influence the response to supplementation. In addition, baseline fiber intake level was not maintained during the intervention by all participants, as both subgroups displayed a drift toward an intermediate value. However, the changes were considered minor deviations to the protocol, and the mean fiber intake in both subgroups remained far apart. Finally, although maltodextrin is commonly used as a placebo in controlled clinical trials related to fiber, it may not be totally neutral for the gut microbiota as shown in a systematic review (51). Nevertheless, maltodextrins are rapidly digested and absorbed, making colonic exposure and microbial fermentation unlikely at the physiological dose used. This limited colonic fermentation could partly explain why a subset of participants (16%) responded to the placebo.

## Conclusion

This study provides evidence that supplementation with RD stimulates the *Parabacteroides* genus within the gut microbiota, no matter the level of dietary fiber intake, but with a stronger effect shown in high fiber consumers. These results further document the potential of prebiotic supplementation with a soluble fiber to shape the gut microbiota as part of a preventive strategy to promote health.

## Data Availability

The datasets presented in this study can be found in the European Nucleotide Archive (https://www.ebi.ac.uk/ena), accession PRJEB108118.
