# Supplementary material for: Resistant dextrin promotes beneficial fecal bacteria in high and low fiber diet populations: a randomized, double-blinded, controlled pilot study
Source: Front Nutr. 2026 May 20;13:1810842. doi: 10.3389/fnut.2026.1810842 (PMC13232062; doi:10.3389/fnut.2026.1810842)
Supplement: Supplementary file 1 [file Table_1.docx]

**Table S1. Composition of Clostridium Cluster IV and Cluster XIVa**

| **ClusterIV** | **ClusterXIVa** |
| --- | --- |
| [Clostridium] leptum | Blautia coccoides |
| [Clostridium] cellulosi | Lacrimispora aerotolerans |
| Flavonifractor plautii | Enterocloster aldenensis |
| [Clostridium] sporosphaeroides | [Clostridium] aminophilum |
| Faecalibacterium prausnitzii | Anaerocolumna aminovalerica |
| Papillibacter cinnamivorans | [Clostridium] asparagiforme |
| Ruminococcus bromii | Enterocloster bolteae |
| Subdoligranulum variabile | [Clostridium] celerecrescens |
|  | Enterocloster citroniae |
|  | Enterocloster clostridioformis |
|  | Enterocloster lavalensis |
|  | Faecalicatena orotica |
|  | [Clostridium] polysaccharolyticum |
|  | Anaeromicropila populeti |
|  | [Clostridium] symbiosum |
|  | Lacrimispora sphenoides |
|  | Lacrimispora xylanolytica |
|  | Acetitomaculum ruminis |
|  | Anaerostipes caccae |
|  | Butyrivibrio crossotus |
|  | Roseburia intestinalis |
|  | Coprococcus eutactus |
|  | Coprococcus comes |
|  | [Ruminococcus] torques |
|  | Mediterraneibacter faecis |
|  | [Ruminococcus] lactaris |
|  | Blautia hansenii |
|  | [Eubacterium] cellulosolvens |
|  | [Eubacterium] rectale |
|  | Eubacterium ventriosum |
|  | Blautia producta |

Source: Hobden MR, Martin-Morales A, Guérin-Deremaux L, Wils D, Costabile A, Walton GE, Rowland I, Kennedy OB, Gibson GR. In Vitro Fermentation of NUTRIOSE® FB06, a Wheat Dextrin Soluble Fibre, in a Continuous Culture Human Colonic Model System. *PLoS ONE* (2013) 8: doi: 10.1371/journal.pone.0077128
