# Supplementary material for: Resistant dextrin promotes beneficial fecal bacteria in high and low fiber diet populations: a randomized, double-blinded, controlled pilot study
Source: Front Nutr. 2026 May 20;13:1810842. doi: 10.3389/fnut.2026.1810842 (PMC13232062; doi:10.3389/fnut.2026.1810842)
Supplement: Supplementary file 2 [file Table_2.docx]

**Table S2. Enzyme commission numbers associated with glucosidase activity and short-chain fatty acid production**

|  | **Enzyme commission number** |
| --- | --- |
| **Glucosidases** |  |
| α | 3.2.1.20 |
| β | 3.2.1.21 |
| **Short chain fatty acids** |  |
| Butyrate | 2.7.2.7 |
|  | 2.8.3.8 |
|  | 2.8.3.9 |
| Propionate | 2.7.2.15 |
|  | 2.8.3.1 |
|  | 2.8.3.27 |
|  | 2.8.3.M4 |
| Acetate | 2.7.2.1 |
|  | 2.8.3.3 |
|  | 2.8.3.8 |
|  | 2.8.3.9 |
|  | 2.8.3.10 |
|  | 2.8.3.11 |
|  | 2.8.3.12 |
|  | 2.8.3.18 |
|  | 2.8.3.19 |
|  | 2.8.3.27 |

Enzyme commission numbers related to short chain fatty acid production were obtained from the BRENDA database (1) and completed from (2).

1. Hauenstein J, Jeske L, Jäde A, Krull M, Dümmer K, Koblitz J, Tietz A, Jahn D, Reimer LC, Bunk B. BRENDA in 2026: a Global Core Biodata Resource for functional enzyme and metabolic data within the DSMZ Digital Diversity. *Nucleic Acids Res* (2025)gkaf1113. doi: 10.1093/nar/gkaf1113

2. Frolova MS, Suvorova IA, Iablokov SN, Petrov SN, Rodionov DA. Genomic reconstruction of short-chain fatty acid production by the human gut microbiota. *Front Mol Biosci* (2022) 9:949563. doi: 10.3389/fmolb.2022.949563
