# Supplementary material for: Resistant dextrin promotes beneficial fecal bacteria in high and low fiber diet populations: a randomized, double-blinded, controlled pilot study
Source: Front Nutr. 2026 May 20;13:1810842. doi: 10.3389/fnut.2026.1810842 (PMC13232062; doi:10.3389/fnut.2026.1810842)
Supplement: Supplementary file 3 [file Table_3.docx]

**Table S3. Dietary fiber intake at each study visit, per treatment arm and subgroup in the ITT population**

| **Variable** |  | **RD** |  |  |  | **PLACEBO** |  |
| --- | --- | --- | --- | --- | --- | --- | --- |
|  | **HF + LF**  **(n=62)** | **HF**  **(n=33)** | **LF**  **(n=29)** |  | **HF + LF**  **(n=62)** | **HF**  **(n=34)** | **LF**  **(n=28)** |
| **V1** |  |  |  |  |  |  |  |
| Mean (SD) | 22.3 (11.5) | 31.0 (8.8) | 12.3 (2.4) |  | 22.4 (11.4) | 30.7 (8.9) | 12.4 (2.1) |
| (Min ; Max) | (5.7 ; 67.4) | (25.1 ; 67.4) | (5.7 ; 15.2) |  | (6.5 ; 67.1) | (25.0 ; 67.1) | (6.5 ; 14.8) |
| Median (Q1 ; Q3) | 25.1 (12.6 ; 28.4) | 27.7 (26.1 ; 30.4) | 12.5 (11.0 ; 14.6) |  | 25.2 (13.0 ; 28.5) | 27.8 (25.6 ; 30.8) | 12.8 (11.4 ; 14.0) |
| **V2** |  |  |  |  |  |  |  |
| Mean (SD) | 22.5 (11.7) | 27.5 (12.1) | 16.7 (8.0) |  | 19.9 (9.2) | 24.6 (9.3) | 14.3 (4.9) |
| (Min ; Max) | (8.1 ; 78.3) | (13.5 ; 78.3) | (8.1 ; 43.6) |  | (4.8 ; 58.8) | (10.8 ; 58.8) | (4.8 ; 28.4) |
| Median (Q1 ; Q3) | 20.8 (13.7 ; 26.6) | 25.2 (21.2 ; 30.2) | 13.7 (12.5 ; 18.3) |  | 18.3 (12.4 ; 25.3) | 24.1 (19.0 ; 28.7) | 13.0 (11.1 ; 15.9) |
| **V3** |  |  |  |  |  |  |  |
| Mean (SD) | 19.2 (7.6) | 22.6 (8.3) | 15.3 (4.0) |  | 20.5 (9.5) | 26.1 (9.1) | 13.7 (3.8) |
| (Min ; Max) | (9.0 ; 53.8) | (13.9 ; 53.8) | (9.0 ; 24.3) |  | (4.9 ; 55.1) | (13.8 ; 55.1) | (4.9 ; 22.0) |
| Median (Q1 ; Q3) | 17.2 (14.5 ; 22.6) | 19.3 (17.2 ; 26.7) | 15.0 (12.8 ; 16.9) |  | 18.2 (14.5 ; 25.3) | 24.7 (19.7 ; 29.3) | 14.4 (11.0 ; 15.7) |

*HF: high dietary fiber group; LF: low dietary fiber group; HF+LF: both groups. RD: resistant dextrin. Data correspond to dietary fiber intake (g/day) and do not include fiber from RD.*
