# Supplementary material for: Resistant dextrin promotes beneficial fecal bacteria in high and low fiber diet populations: a randomized, double-blinded, controlled pilot study
Source: Front Nutr. 2026 May 20;13:1810842. doi: 10.3389/fnut.2026.1810842 (PMC13232062; doi:10.3389/fnut.2026.1810842)
Supplement: Supplementary file 4 [file Table_4.docx]

**Table S4: Relative abundance of the Parabacteroides genus in high (HF) and low (LF) dietary fiber subgroups and in the whole cohort (HF+LF) per study arm in the ITT population**

| Visit | Fiber group |  | RD  (n=62) | Placebo  (n=62) | All  (n=124) |
| --- | --- | --- | --- | --- | --- |
| V1 | LF | N | 27 | 28 | 55 |
|  |  | Missing | 2 | 0 | 2 |
|  |  | Prevalence (%) | 100 | 100 | 100 |
|  |  | Mean (SD) | 1.691 (1.941) | 1.321 (1.103) | 1.509 (1.583) |
|  |  | (Min ; Max) | (0.11; 9.181) | (0.082; 3.606) | (0.082; 9.181) |
|  |  | Median (Q1 ; Q3) | 0.949 (0.461; 2.575) | 1.039 (0.374; 1.946) | 1.039 (0.402; 2.201) |
|  | HF | N | 32 | 33 | 65 |
|  |  | Missing | 1 | 1 | 2 |
|  |  | Prevalence (%) | 100 | 100 | 100 |
|  |  | Mean (SD) | 1.112 (1.497) | 1.408 (1.278) | 1.265 (1.386) |
|  |  | (Min ; Max) | (0.082; 8.559) | (0.142; 5.777) | (0.082; 8.559) |
|  |  | Median (Q1 ; Q3) | 0.781 (0.342; 1.412) | 1.079 (0.702; 1.594) | 0.886 (0.563; 1.544) |
|  | LF+HF | N | 59 | 61 | 120 |
|  |  | Missing | 3 | 1 | 4 |
|  |  | Prevalence (%) | 100 | 100 | 100 |
|  |  | Mean (SD) | 1.382 (1.728) | 1.369 (1.195) | 1.376 (1.477) |
|  |  | (Min ; Max) | (0.082; 9.181) | (0.082; 5.777) | (0.082; 9.181) |
|  |  | Median (Q1 ; Q3) | 0.806 (0.365; 1.658) | 1.074 (0.58; 1.67) | 0.931 (0.5; 1.67) |
| V2 | LF | N | 28 | 27 | 55 |
|  |  | Missing | 1 | 1 | 2 |
|  |  | Prevalence (%) | 100 | 100 | 100 |
|  |  | Mean (SD) | 5.458 (5.124) | 1.344 (1.222) | 3.438 (4.261) |
|  |  | (Min ; Max) | (0.096; 22.274) | (0.058; 4.657) | (0.058; 22.274) |
|  |  | Median (Q1 ; Q3) | 3.836 (1.645; 8.256) | 0.97 (0.42; 1.847) | 1.808 (0.849; 4.154) |
|  | HF | N | 32 | 34 | 66 |
|  |  | Missing | 1 | 0 | 1 |
|  |  | Prevalence (%) | 100 | 100 | 100 |
|  |  | Mean (SD) | 5.474 (4.793) | 1.511 (1.242) | 3.432 (3.965) |
|  |  | (Min ; Max) | (0.837; 19.756) | (0.132; 6.008) | (0.132; 19.756) |
|  |  | Median (Q1 ; Q3) | 3.834 (2.196; 6.561) | 1.094 (0.722; 2.167) | 2.14 (0.915; 4.07) |
|  | LF+HF | N | 60 | 61 | 121 |
|  |  | Missing | 2 | 1 | 3 |
|  |  | Prevalence (%) | 100 | 100 | 100 |
|  |  | Mean (SD) | 5.466 (4.908) | 1.437 (1.226) | 3.435 (4.085) |
|  |  | (Min ; Max) | (0.096; 22.274) | (0.058; 6.008) | (0.058; 22.274) |
|  |  | Median (Q1 ; Q3) | 3.836 (1.985; 8.239) | 1.068 (0.523; 1.979) | 1.979 (0.872; 4.154) |
| V3 | LF | N | 28 | 28 | 56 |
|  |  | Missing | 1 | 0 | 1 |
|  |  | Prevalence (%) | 100 | 100 | 100 |
|  |  | Mean (SD) | 1.249 (0.779) | 1.599 (2.129) | 1.424 (1.598) |
|  |  | (Min ; Max) | (0.25; 3.612) | (0.113; 7.989) | (0.113; 7.989) |
|  |  | Median (Q1 ; Q3) | 1.078 (0.697; 1.624) | 0.958 (0.274; 1.654) | 1.026 (0.541; 1.624) |
|  | HF | N | 29 | 33 | 62 |
|  |  | Missing | 4 | 1 | 5 |
|  |  | Prevalence (%) | 100 | 100 | 100 |
|  |  | Mean (SD) | 1.032 (0.778) | 1.394 (1.209) | 1.225 (1.038) |
|  |  | (Min ; Max) | (0.047; 3.409) | (0.166; 5.953) | (0.047; 5.953) |
|  |  | Median (Q1 ; Q3) | 0.859 (0.507; 1.17) | 0.978 (0.623; 1.719) | 0.903 (0.558; 1.598) |
|  | LF+HF | N | 57 | 61 | 118 |
|  |  | Missing | 5 | 1 | 6 |
|  |  | Prevalence (%) | 100 | 100 | 100 |
|  |  | Mean (SD) | 1.139 (0.779) | 1.488 (1.682) | 1.319 (1.331) |
|  |  | (Min ; Max) | (0.047; 3.612) | (0.113; 7.989) | (0.047; 7.989) |
|  |  | Median (Q1 ; Q3) | 0.997 (0.615; 1.503) | 0.978 (0.462; 1.719) | 0.984 (0.551; 1.62) |

*ITT: intention to treat population; HF: high dietary fiber group; LF: low dietary fiber group; HF+LF: both groups. RD: resistant dextrin. Values are relative abundance (%) within bacterial genera of the fecal microbiota*
