# Supplementary material for: Resistant dextrin promotes beneficial fecal bacteria in high and low fiber diet populations: a randomized, double-blinded, controlled pilot study
Source: Front Nutr. 2026 May 20;13:1810842. doi: 10.3389/fnut.2026.1810842 (PMC13232062; doi:10.3389/fnut.2026.1810842)
Supplement: Supplementary file 5 [file Table_5.docx]

**Table S5. Inferential analysis of the mean stool frequency in the ITT population**

| **Visit** | **Fiber Group** |  | **N** | **Rate estimate** | **Rate Ratio** | **95% confidence interval** | **P value** |
| --- | --- | --- | --- | --- | --- | --- | --- |
| V2 | HF | Placebo | 34 | 1.657 |  | [1.47;1.86] |  |
|  | HF | RD | 32 | 1.640 |  | [1.45;1.86] |  |
|  | HF | RD vs Placebo |  |  | 0.99 | [0.84;1.17] | 0.9048 |
|  | LF | Placebo | 28 | 1.563 |  | [1.37;1.79] |  |
|  | LF | RD | 28 | 1.749 |  | [1.53;2.00] |  |
|  | LF | RD vs Placebo |  |  | 1.12 | [0.93;1.34] | 0.2181 |
|  | HF+LF | Placebo |  | 1.609 |  | [1.47;1.76] |  |
|  | HF+LF | RD |  | 1.694 |  | [1.54;1.87] |  |
|  | HF+LF | RD vs Placebo |  |  | 1.05 | [0.93;1.19] | 0.4161 |
|  | LF vs HF | RD - Placebo |  |  | 1.13 | [0.89;1.44] | 0.3193 |
|  |  |  |  |  |  |  |  |
| V3 | HF | Placebo | 34 | 1.667 |  | [1.48;1.87] |  |
|  | HF | RD | 32 | 1.584 |  | [1.40;1.80] |  |
|  | HF | RD vs Placebo |  |  | 0.95 | [0.80;1.12] | 0.5471 |
|  | LF | Placebo | 28 | 1.429 |  | [1.24;1.64] |  |
|  | LF | RD | 28 | 1.678 |  | [1.46;1.92] |  |
|  | LF | RD vs Placebo |  |  | 1.17 | [0.98;1.41] | 0.0887 |
|  | HF+LF | Placebo |  | 1.544 |  | [1.41;1.69] |  |
|  | HF+LF | RD |  | 1.630 |  | [1.48;1.80] |  |
|  | HF+LF | RD vs Placebo |  |  | 1.06 | [0.93;1.20] | 0.3942 |
|  | LF vs HF | RD - Placebo |  |  | 1.24 | [0.96;1.58] | 0.0927 |
|  |  |  |  |  |  |  |  |
|  |  | Product |  |  |  |  | 0.2668 |
|  |  | Visit |  |  |  |  | 0.3429 |
|  |  | Fiber Group |  |  |  |  | 0.6244 |
|  |  | Product*Visit |  |  |  |  | 0.9667 |
|  |  | Product*Fiber Group |  |  |  |  | 0.0687 |
|  |  | Visit*Fiber Group |  |  |  |  | 0.5446 |
|  |  | Product*Visit*Fiber Group |  |  |  |  | 0.5949 |
|  |  | Baseline |  |  |  |  | <0.0001 (***) |

*ITT: intention to treat population;* *HF: high dietary fiber group; LF: low dietary fiber group; HF+LF: both groups. RD: resistant dextrin. Stool frequency was recorded for 7 days before the visit on a diary.*

*Results are obtained from a Mixed Poisson regression model (random effect on site was removed due to non-convergence). The rate estimate corresponds to the number of stools per day as estimated by the model. The rate ratio is calculated between arms (RD versus placebo) or between dietary fiber groups (LF versus HF) for the difference of stool frequency between RD and placebo.*
